# Flow-dependent myosin recruitment during *Drosophila* cellularization requires zygotic *dunk* activity

**DOI:** 10.1242/dev.131334

**Published:** 2016-07-01

**Authors:** Bing He, Adam Martin, Eric Wieschaus

**Affiliations:** 1 Department of Molecular Biology, Princeton University, Princeton, NJ 08544, USA; 2 HHMI, Princeton University, Princeton, NJ 08544, USA

**Keywords:** Cytokinesis, Cellularization, Cortical myosin recruitment, Actomyosin network, *Dunk*

## Abstract

Actomyosin contractility underlies force generation in morphogenesis ranging from cytokinesis to epithelial extension or invagination. In *Drosophila*, the cleavage of the syncytial blastoderm is initiated by an actomyosin network at the base of membrane furrows that invaginate from the surface of the embryo. It remains unclear how this network forms and how it affects tissue mechanics. Here, we show that during *Drosophila* cleavage, myosin recruitment to the cleavage furrows proceeds in temporally distinct phases of tension-driven cortical flow and direct recruitment, regulated by different zygotic genes. We identify the gene *dunk*, which we show is transiently transcribed when cellularization starts and functions to maintain cortical myosin during the flow phase. The subsequent direct myosin recruitment, however, is Dunk-independent but requires Slam. The Slam-dependent direct recruitment of myosin is sufficient to drive cleavage in the *dunk* mutant, and the subsequent development of the mutant is normal. In the *dunk* mutant, cortical myosin loss triggers misdirected flow and disrupts the hexagonal packing of the ingressing furrows. Computer simulation coupled with laser ablation suggests that Dunk-dependent maintenance of cortical myosin enables mechanical tension build-up, thereby providing a mechanism to guide myosin flow and define the hexagonal symmetry of the furrows.

## Abstract (precis)

**Summary:** During *Drosophila* cellularisation, myosin recruitment to the cleavage furrows proceeds in temporally and mechanistically distinct phases separately regulated by *dunk* and *slam*.

## INTRODUCTION

Contraction of filamentous actin networks by non-muscle myosin II (hereafter ‘myosin’) provides a widely used mechanism for force generation during cell and tissue morphogenesis (Munjal and Lecuit, 2014; Martin and Goldstein, 2014). For example, a variety of morphogenetic processes ranging from cytokinesis to tissue spreading and elongation are driven by constriction of contractile actomyosin rings (Green et al., 2012; Martin and Lewis, 1992; Hutson et al., 2003; Behrndt et al., 2012; Sehring et al., 2014). Recruitment of myosin to specific regions of the cell cortex is a crucial step for the subsequent assembly of contractile actomyosin structures and essentially determines the spatiotemporal distribution of forces. Studies in various organisms suggest two general strategies for this recruitment. In the first mechanism, myosin filaments are assembled and recruited to relatively broad regions of the cell cortex and subsequently undergo motor-dependent flow (‘cortical flow’). Myosin flows are thought to be powered by asymmetrical contraction and have been demonstrated in many actomyosin-based processes, such as cytokinesis (DeBiasio et al., 1996; Yumura et al., 2008; Uehara et al., 2010), embryo polarity establishment (Munro et al., 2004), convergent extension (Rauzi et al., 2010), cell sheet spreading (Behrndt et al., 2012) and apical constriction (Munjal et al., 2015). The second mechanism suggests direct recruitment of myosin filaments to the designated regions of the cell cortex without undergoing cortical flow (‘direct recruitment’; Zang and Spudich, 1998; Yumura et al., 2008; Zhou and Wang, 2008; Vale et al., 2009; Beach and Egelhoff, 2009; Ma et al., 2012). These mechanisms appear to function redundantly in different systems to ensure the proper assembly of the actomyosin contractile machineries. However, the timing and extent of the contribution of each of the mechanisms to myosin recruitment and localization remain elusive.

**Fig. 1.**
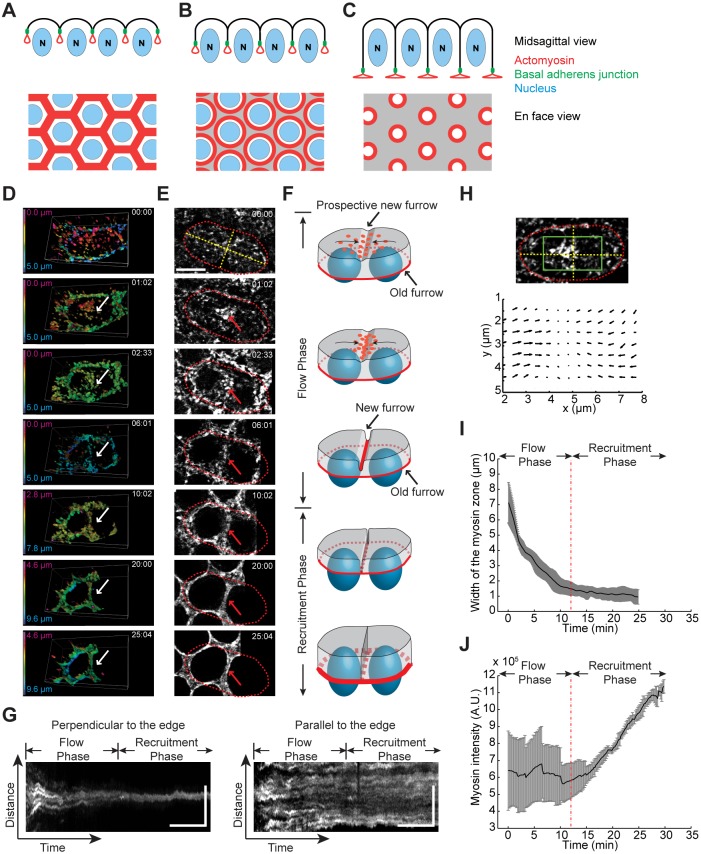
**Biphasic recruitment of myosin to the invagination front during cellularization.** (A-C) Schematics showing the midsagittal view (top) and the en face view (bottom) of a cellularizing embryo. Red, actomyosin; blue, nucleus; green, basal adherens junction. Actin and myosin first accumulate at the invagination front and form a hexagonal network (A). As cellularization proceeds, the actomyosin network reorganizes into individual actomyosin rings (B) which then constrict to close the basal side of the newly formed cells (C). (D) 3D rendering of Sqh-GFP movies demonstrating cortical myosin flow during early cellularization. Shown is a single pair of daughter nuclei. Color coding corresponds to the depth of myosin structures from the apical surface. (E) Projections of Sqh-GFP at the invagination front that correspond to the 3D images in D. Red dotted lines highlight the outline of the two daughter nuclei. Yellow dotted lines correspond to the axes that are parallel or perpendicular to the newly formed edge between the two daughter nuclei. Arrows in D and E indicate the position of the new furrow. Scale bar: 5 µm. (F) Schematics demonstrating the separate phases of myosin recruitment during cellularization. Red, myosin; blue, nucleus. Arrows on the apical surface indicate the direction of myosin flow. (G) Kymographs corresponding to the yellow lines in E, showing myosin flow perpendicular (left) or parallel (right) to the edge. Scale bars: 5 min (*x*); 5 µm (*y*). (H) An example of particle image velocimetry (PIV) analysis of cortical Sqh-GFP during the flow phase. The velocity map corresponds to the boxed region in the image above. (I) Measurement of the width of the myosin band at newly formed edges (*n*=4 edges). Gray area indicates ±s.d. (J) Measurement of total myosin intensity at the invagination front (*n*=3 embryos). Gray area indicates ±s.d.

*Drosophila* cellularization, an atypical cytokinesis that cleaves the syncytial embryos, provides a unique system to study the assembly and reorganization of actomyosin structures. Before cellularization, the embryo undergoes 13 nuclear divisions without cytokinesis, resulting in a syncytium with ∼5000 nuclei spread out at the periphery of the embryo. At the beginning of interphase cycle 14, membrane furrows invaginate from the surface of the embryo and form a honeycomb-like hexagonal array, with each hexagonal unit enclosing one nucleus (Mazumdar and Mazumdar, 2002). As soon as the membrane furrows start to invaginate, actin and myosin accumulate at the invagination front and form a network (Fig. 1A-C; Kiehart, 1990; Young et al., 1991; Schejter and Wieschaus, 1993; Field and Alberts, 1995; Foe et al., 2000; Royou, et al., 2004). As furrow ingression proceeds, the actomyosin network reorganizes into individual actomyosin rings, which then constrict to close the basal side of the newly formed cells in a manner resembling typical animal cytokinesis (Schejter and Wieschaus, 1993). Although the initial geometries are different, a number of proteins involved in cytokinesis also function in cellularization, such as the scaffolding proteins Scraps (Anillin homolog; Field et al., 2005) and Septins (Adam et al., 2000), the small GTPase Rho1 (RhoA homolog; Crawford et al., 1998), and the formin-family actin nucleator Diaphanous (Dia; Afshar et al., 2000; Grosshans et al., 2005), to name a few.

*Drosophila* cellularization also requires additional regulation apart from that in typical cytokinesis. Although most proteins functioning in cellularization are maternally provided, the timing of cellularization is critically controlled by a small set of zygotic genes that does not function in post-cellularization cytokinesis. Previous genome-wide deficiency screens have identified a small number of genomic regions that are zygotically required for cellularization (Merrill et al., 1988; Wieschaus and Sweeton, 1988). Several genes have been subsequently cloned. Four of them, *bottleneck* (*bnk*), *nullo*, *Serendipity α* (*Sry-α*) and *slam*, are specifically expressed during cellularization and regulate the organization of the actomyosin network (Schweisguth et al., 1990; Schejter and Wieschaus, 1993; Postner and Wieschaus, 1994; Lecuit et al., 2002; Stein et al., 2002; Grosshans et al., 2005; Sokac and Wieschaus, 2008a; Wenzl et al., 2010; Zheng et al., 2013). Despite the identification of these genes, it remains unclear how myosin is recruited to the invagination front and how the actomyosin network is established and regulated.

Here, we demonstrate that the recruitment of myosin during *Drosophila* cellularization proceeds in two temporally distinct phases. First, a tension-driven cortical flow brings myosin to the leading edge of the cleavage furrows. Subsequently, additional myosin is directly recruited from the cytoplasm to the leading edge. The myosin flow is anisotropic and is similar to the tension-based flow that drives myosin into the cleavage furrow during typical cytokinesis. By cloning and characterizing a cellularization-specific gene *dunk*, we demonstrate that the cortical flow-mediated myosin recruitment requires a Dunk-dependent mechanism that prevents myosin loss from the cortex. We also present genetic evidence that the direct recruitment of myosin in the second phase is Dunk-independent but requires Slam. Our findings demonstrate that separate myosin recruitment mechanisms are developmentally modulated by different zygotic genes to regulate cellularization in a coordinated manner.

## RESULTS

### Biphasic recruitment of myosin to the leading edge of the cleavage furrows during cellularization

In order to elucidate how myosin is recruited to the invagination front during cellularization, we made high-resolution, time-lapse movies of myosin using embryos expressing Sqh-GFP (myosin regulatory light chain fused to GFP; Royou et al., 2002; Movies 1 and 2). Fig. 1D shows the 3D rendering of Sqh-GFP during early and mid-cellularization with the myosin structures pseudocolor-coded corresponding to their depth from the apical surface. Fig. 1E shows the corresponding projections at the invagination front. At the transition between telophase 13 and interphase 14, myosin first appears at the base of the retracting metaphase furrows (the old furrows) along the circumference of the previous mitotic figure, ∼5 µm below the apical surface (shown as ‘blue myosin’ in Fig. 1D at *t*=0 min; see Fig. 1F for an illustration of the old and new furrows). Myosin puncta appear at the apical cortex approximately 1 min later (shown as ‘magenta myosin’ in Fig. 1D at *t*=0 min), and are slightly more enriched near the prospective furrow (the new furrow) between the two corresponding daughter nuclei. As that furrow begins to invaginate, myosin puncta flow towards and become enriched at the base of the furrow (Fig. 1D,E, arrows). To illustrate the myosin flow better, we generated kymographs in the direction either perpendicular or parallel to the newly formed furrow (Fig. 1G). In the direction perpendicular to the furrow, myosin trajectories converge towards the furrow (Fig. 1G, left), whereas in the direction parallel to the furrow, myosin trajectories remain parallel to each other (Fig. 1G, right). These results demonstrate that myosin predominantly moves in the direction perpendicular to the edge but not parallel to the edge. We further examined the velocity distribution of myosin flow using particle image velocimetry (PIV) analysis. The result confirmed the strong velocity anisotropy biased towards the direction parallel to the furrow (Fig. 1H).

The onset of apical myosin flow coincides with the formation of the new furrow, which in our experiments we define as the beginning of cellularization (*t*=0). At *t*=4-5 min, the old and new furrows reach the same depth of ∼3 µm from the surface of the embryo and become nearly indistinguishable (Fig. 1D,E; Fig. S1). At this point, myosin at the base of old and new furrows appears to join and forms an interconnected network across the entire invagination front (Fig. 1E at *t*=6 min). At *t*=20 min, as the invagination front passes approximately one-third of nuclei length, the actomyosin network starts to reorganize into individual rings surrounding each nucleus (Fig. 1E at *t*=20 min). These rings become well resolved at *t*=30 min, as furrow ingression transitions from a slow-growing to a fast-growing phase (Merrill et al., 1988; Lecuit and Wieschaus, 2000; Figard et al., 2013).

During the first 12 min after the onset of cellularization, as myosin flows continuously towards the base of the furrows, the interfaces of the myosin network separating adjacent nuclei (which we call ‘edges’) narrow in width (Fig. 1I), whereas the total myosin intensity in the forming network plus apical cortex remains constant (Fig. 1J). During the next 20 min, the width of edges no longer changes (Fig. 1I), but total myosin intensity increases (Fig. 1J). These observations identify two temporally distinct phases in myosin recruitment to the invagination front. In the first phase, the myosin puncta present at the apical cortex undergo a cortical flow towards the base of the ingressing furrow (henceforth ‘the flow phase’). In the second phase, new myosin appears to be directly recruited from the cytoplasm to the invagination front without cortical flow (henceforth ‘the recruitment phase’). Therefore, during *Drosophila* cellularization, myosin cortical flow and direct myosin recruitment are used at distinct times to recruit myosin to the base of the newly formed furrows (Fig. 1F).

### The actomyosin network at the invagination front is under tension

**Fig. 2.**
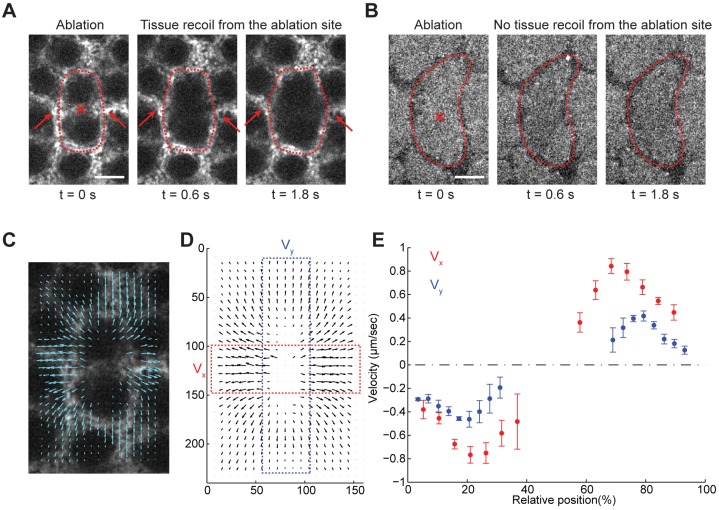
**The actomyosin network is under tension.** (A) Laser ablation of a newly formed cleavage furrow in an embryo expressing Sqh-GFP. Red dotted lines mark the outline of the previous mitotic figure. The red cross marks the site of laser ablation. Arrows highlight the retraction of tissues from the incision site. Scale bar: 5 µm. (B) Laser ablation at the apical cortex in an embryo at cycle 13 anaphase. Red dotted lines mark the outline of the mitotic figure. Scale bar: 5 µm. (C) An example of PIV analysis of Sqh-GFP immediately after laser ablation. The velocity map corresponds to the images shown in A. (D) Average velocity map immediately after laser ablation. *N*=24 ablations in eight embryos. (E) Average velocity distribution along the boxed regions in D. Error bars indicate s.d.

The flow of myosin during early cellularization is reminiscent of the tension-driven myosin flows thought to play a role in contractile ring formation during cytokinesis. To test whether the cortex is under tension, we used a focused UV laser beam to ablate the invagination front in flow-phase embryos. If the cortex is under tension, the surrounding tissues will undergo recoil, and the initial velocity of recoil is proportional to the resting tension divided by the viscous drag, which is assumed to be constant between experiments (Hutson et al., 2003; Martin et al., 2010). Single spot incision in the middle of an edge resulted in an immediate displacement of the surrounding tissues away from the incision site (Fig. 2A, tissue movement is indicated by arrows; Movie 3). This tension appears to arise at the beginning of cycle 14 simultaneous with myosin recruitment to the surface, as laser incision made at the apical cortex before this time point did not induce appreciable tissue recoil (Fig. 2B, as indicated by lack of changes in the apical cortex; Movie 3). These results suggest that tension at the invagination front is due to actomyosin contractility.

If the tension and myosin flow described above is analogous to motor-dependent myosin flow in cytokinesis, it will move and align cytoskeletal elements, thereby accounting for the narrowing of the myosin band. PIV analysis demonstrated that the movement of the surrounding tissue after laser ablation is anisotropic. As demonstrated in Fig. 2C,D and quantified in Fig. 2E, the velocity vectors parallel to the ablated edge (V_x_) are larger than those perpendicular to the edge (V_y_). This anisotropy in tissue tension is an expected pattern because if the initial broad contractile network drives a flow that is perpendicular to the edge, tension in that direction will be released. The interconnectedness of the forming network would not allow flow between vertices (Fig. 1F,G) and thus tensions will remain high in directions parallel to the edge. In the following sections, we present genetic and molecular evidence that the separate phases of myosin recruitment require distinct zygotic gene activities and that the anisotropy of the myosin flow requires maintenance of myosin at the cortex that is developmentally regulated by a novel gene *dunk*.

### Mutation in *dunk* causes cortical myosin loss specifically during the flow phase

In a genome-wide mapping and transcriptional profiling study of *Drosophila* heterochromatin (He et al., 2012), we identified *dunk* (CG34137; FlyBase Genome Annotators, 2006), a blastoderm stage-specific gene located near the centromere of the second chromosome. *dunk* is an intron-less gene that encodes a 246-amino-acid-long protein with no previously characterized homologs or well-defined structural motifs (data not shown). *dunk* has close homologs in several other *Drosophila* species and a more distant homolog in house flies, but has no obvious homologs in other species. Interestingly, we identified two conserved binding sites for the zinc-finger transcriptional activator Zelda near the transcription start site of *dunk*, a feature shared by many Zelda-dependent early transcribed zygotic genes in *Drosophila* (Liang et al., 2008).

**Fig. 3.**
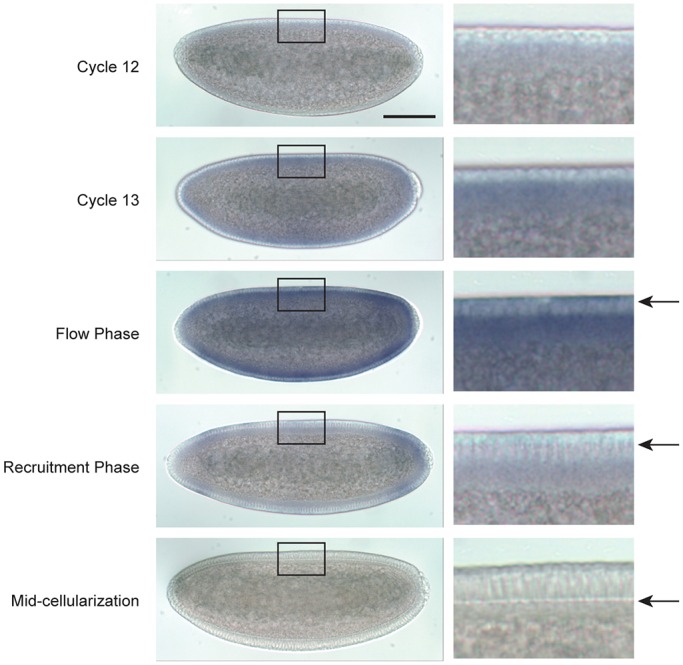
***dunk* is transiently transcribed at the onset of cellularization.** *In situ* hybridization of wild-type embryos with an antisense *dunk* probe. Note that *dunk* is transiently induced immediately before cellularization starts and rapidly diminishes during late cellularization. Arrows indicate the depth of invagination font. Boxed regions are enlarged to the right. Scale bar: 100 µm.

*In situ* hybridization demonstrates that *dunk* transcripts are not present in pre-syncytial embryos and only become detectable at cycle 13 (Fig. 3). *dunk* transcripts peak in early cellularization, are distributed uniformly across the whole embryo, and then rapidly diminish during late cellularization. We identified a P-element insertion allele of *dunk* generated by the Berkeley *Drosophila* Genome Project gene disruption project (Bellen et al., 2004; henceforth *dunk^1^*). The *dunk^1^* allele is predicted to generate truncated proteins that lack the C-terminal three-quarters of the normal sequence. No transcript was detected in embryos homozygous for *dunk^1^*, suggesting that this mutant is a protein null (see below).

**Fig. 4.**
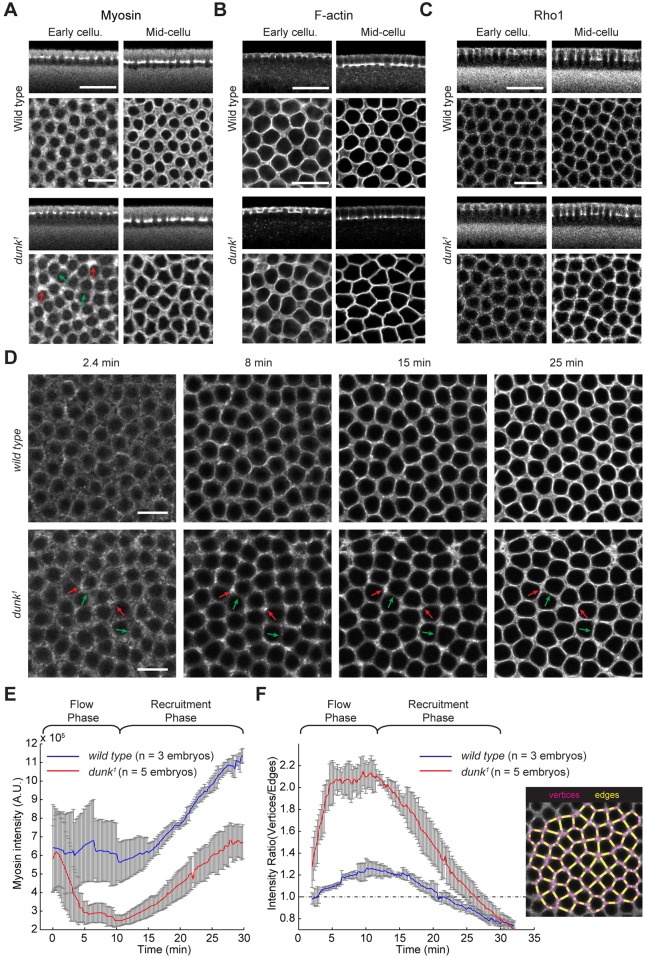
***dunk^1^* mutant embryos fail to maintain myosin at the cortex during the flow phase.** (A-C) Immunostaining (A,C) or phalloidin staining (B) showing localization of myosin (Zipper) (A), F-actin (B) and Rho1 (C) in cross-sections (top) and projections of confocal sections at the invagination front (bottom). In *dunk^1^* mutant embryos at early cellularization, myosin shows inhomogeneous distribution at the invagination front, being preferentially enriched at the vertices (red arrows) but depleted from the edges (green arrows). By contrast, the distributions of F-actin (B) and Rho1 (C) at the invagination front remain homogeneous. During mid-cellularization, although actin and myosin can still form individual rings, the rings are less rounded and frequently highly angular. Scale bars: 25 µm (top); 10 µm (bottom). (D) Projections of confocal sections showing Sqh-GFP at the invagination front in wild-type and *dunk^1^* mutant embryos over time. Note that myosin distribution is abnormally inhomogeneous in *dunk^1^* mutant embryos at *t*=8 min. Myosin preferentially accumulates at vertices (red arrows) and is depleted from edges (green arrows). Scale bars: 10 µm. (E) Quantification of total myosin intensity at the invagination front. Error bars indicate s.d. (F) Left panel: ratio between vertex- and edge-myosin intensities in wild-type (blue) and *dunk^1^* mutant (red) embryos. Error bars indicate s.d. Right panel: schematic showing quantification of myosin intensity at vertices (magenta) and edges (yellow).

*dunk^1^* mutant embryos show defects in myosin organization shortly after the onset of cellularization. In wild-type embryos, myosin is evenly distributed across the invagination front and forms a network. By contrast, in *dunk^1^* homozygous mutant embryos, myosin distribution becomes inhomogeneous, with myosin preferentially accumulating at the vertices (Fig. 4A, red arrows) and being depleted from many of the edges (Fig. 4A, green arrows; hence the name *disrupted underground network*). By contrast, F-actin and Rho1 remain homogeneously distributed at the invagination front with no obvious reduction in protein levels (Fig. 4B,C, the early cellularization panels). Therefore, the myosin defect observed in *dunk^1^* mutants is not likely to be due to loss or redistribution of F-actin or Rho1. At later stages, the distributions of myosin, F-actin and Rho1 all become abnormal in *dunk^1^* mutants (Fig. 4B, the mid-cellularization panels), which probably reflects the defect in the morphology of the invagination front (see below).

To illuminate how the myosin phenotype arises in *dunk^1^* mutant embryos, we examined Sqh-GFP in live embryos (Fig. 4D-F; Movie 4). At the beginning of cellularization, the initial cortical recruitment of myosin is similar in wild-type and *dunk^1^* mutant embryos (Fig. 4D, *t*=2.4 min). In the flow phase, however, myosin rapidly becomes depleted from most edges but remains at vertices and short edges, consistent with the observation in fixed embryos (Fig. 4D, *t*=8 min). Quantification of myosin intensity at the invagination front demonstrated that the drop in cortical myosin intensity occurs within the first 5 min and myosin remains low throughout the flow phase (Fig. 4E). During the recruitment phase, however, myosin intensity increases at a rate comparable to that in wild type (Fig. 4E). To illustrate the biased myosin distribution towards vertices in *dunk^1^* mutants, we plotted the ratio of myosin density at vertices to that at edges (Fig. 4F). In wild type, the ratio remains close to 1. By contrast, in *dunk^1^* mutant embryos, the ratio quickly increases to >2 and remains at the peak during the flow phase. The ratio then gradually returns to 1 during the recruitment phase as new myosin is recruited to the invagination front (Fig. 4F). Taken together, these results suggest that Dunk is specifically required for maintenance of cortical myosin during the flow phase. In the subsequent recruitment phase, a Dunk-independent process appears to function to recruit additional myosin to the invagination front.

The loss of cortical myosin seen in *dunk* mutant embryos might result from alterations in the rate of myosin recruitment and dissociation to and from the cortex. We tested this possibility by measuring the rate of fluorescence recovery after photobleaching (FRAP) on cortical Sqh-GFP (Fig. S2A; Movie 5). Interestingly, we found that the rate of myosin fluorescence recovery in *dunk* mutant embryos is identical to that in wild type, as long as the FRAP recovery is adjusted to the decreasing levels of myosin in the mutant at the time of FRAP (half recovery time in *dunk* mutants is 70.5±15.0 s, mean±s.d., compared with 71.2±18.4 s in wild type; Fig. S2B-D). Therefore, Dunk does not appear to directly regulate the rate of myosin turnover at the cortex and probably promotes cortical myosin stability through other mechanisms.

### Myosin flow is misdirected in *dunk* mutant embryos

**Fig. 5.**
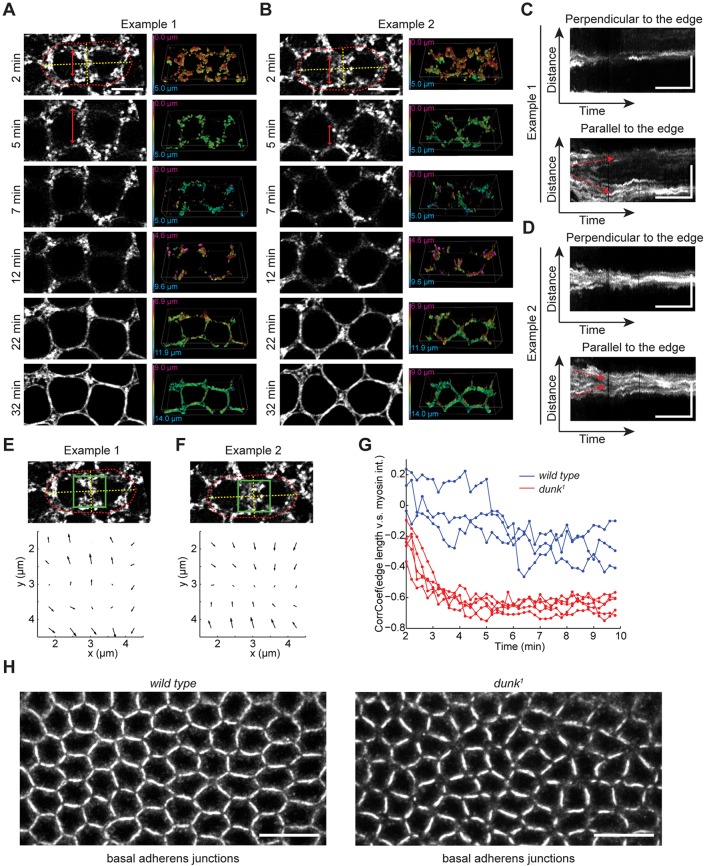
**Myosin flow is destabilized in *dunk^1^* mutant embryos.** (A,B) Two examples demonstrating distinct myosin behaviors at newly formed edges in *dunk^1^* mutant embryos. In each example, the left panel shows projections of confocal sections at the invagination front, and the right panel shows the 3D rendering of the corresponding Sqh-GFP movies. Color coding corresponds to the depth of the myosin structures from the apical surface. Red dotted lines outline the two daughter nuclei. Yellow dotted lines correspond to the axes that are parallel and perpendicular to the newly formed edge between the two daughter nuclei. Arrows in A and B demonstrate the stretching and shortening of the new furrow, respectively. Scale bars: 5 µm. (C,D) Kymographs corresponding to the yellow lines in A and B, respectively. Arrows highlight the divergence (C) or convergence (D) of myosin trajectories in the direction parallel to the new furrow. Scale bars: 5 min (*x*); 5 µm (*y*). (E,F) PIV analysis of Sqh-GFP movement shown in A and B, respectively. The velocity map corresponds to the boxed region in the image above. (G) Correlation between the length of edge and the myosin intensity along the edge at different times after the onset of cellularization. Wild type: *n*=3 embryos; *dunk^1^* mutant: 5 embryos. (H) Immunostaining of Armadillo (*Drosophila* β-catenin homolog) showing the distribution of basal adherens junctions in wild-type and *dunk^1^* mutant embryos at early cellularization. Scale bars: 10 µm.

In wild-type embryos throughout the flow phase, myosin flow is perpendicular to the furrow, and within each edge myosin does not flow parallel to the edge (Fig. 1G,H). This flow pattern tightens the network, while maintaining global network architecture and a relatively uniform distribution of myosin along each edge. In *dunk^1^* embryos, however, the flow of cortical myosin is no longer restricted to the direction perpendicular to the edges (Fig. 5A-F; Movies 6, 7). In most cases (as represented in Fig. 5A,C,E), myosin flows towards the neighboring vertices and becomes depleted from the center of the edge. In the remaining cases (as represented in Fig. 5B,D,F), myosin remains on the edge and flows towards the center. Kymograph analysis shows that in the direction parallel to the edge, the myosin trajectories are no longer parallel to each other as in the wild type (Fig. 1G), but instead either diverge from (Fig. 5C) or converge towards (Fig. 5D) the center of the edge. PIV analysis also demonstrates that the velocity vectors of myosin flow become largely parallel to the edge, either pointing away from (Fig. 5E) or pointing towards (Fig. 5F) the center of the edge.

The flow of myosin parallel to the edge can have one of two consequences on the morphology of the edge. When myosin flows towards the neighboring vertices, the edge stretches in length (Fig. 5A). By contrast, when myosin flows towards the center of the edge, the edge undergoes a contraction and effective shortening (Fig. 5B). As a result, the length of edge and the myosin intensity along that edge becomes negatively correlated (i.e. short borders have more myosin; Fig. 5G). The changes in edge length disrupt the hexagonal symmetry of the ingressing furrows, resulting in an irregular network composed of angular units as shown by staining of basal adherens junctions (Fig. 5H). The actomyosin rings subsequently formed also acquire irregular shapes (Fig. 4A, the mid-cellularization panels). Together, these observations suggest that the biased accumulation of myosin at vertices and the irregular packing geometry of the ingressing furrows seen in *dunk^1^* mutants is a direct consequence of the misdirected myosin flow that occurs coincident with cortical myosin loss.

### A Dunk-dependent mechanical mechanism that guides the anisotropic cortical myosin flow

**Fig. 6.**
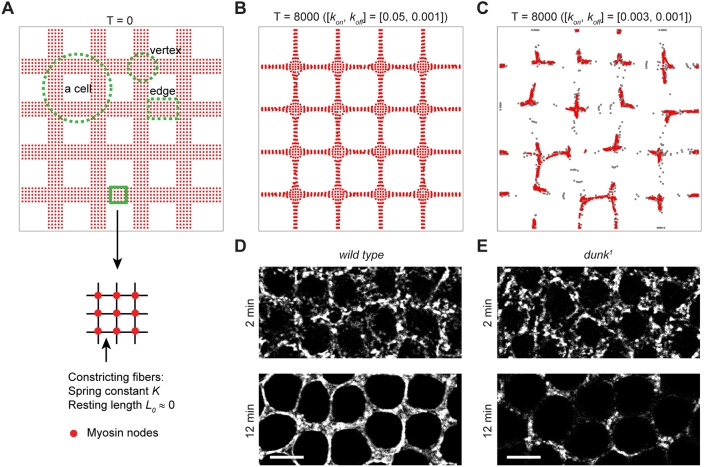
**Computer simulation demonstrates the link between cortical myosin dynamics and the anisotropy of the myosin flow.** (A) Implementation of an interconnected contractile network. The contractile force is applied along each interface between neighboring myosin nodes. Within the network, myosin nodes undergo dynamic turnover with a recruitment rate constant of *k_on_* and a dissociation rate constant of *k_off_*. (B) The model configuration at *t*=8000 simulation steps when *k_on_*≫*k_off_* ([*k_on_*, *k_off_*]=[0.05, 0.001]). Myosin nodes undergo anisotropic flow similar to that observed in the wild-type embryos. (C) The model configuration at *t*=8000 simulation steps when *k_on_*/*k_off_*∼1 ([*k_on_*, *k_off_*]=[0.003, 0.001]). The simulation recapitulates the destabilized myosin flow and vertical accumulation of myosin observed in *dunk^1^* mutant embryos. (D,E) Projections of confocal sections showing Sqh-GFP at the invagination front in a wild-type (D) and *dunk^1^* mutant (E) embryo at *t*=2 min (top) and *t*=12 min (bottom). Scale bars: 5 µm.

What is the mechanistic link between Dunk-dependent maintenance of cortical myosin and the anisotropy of the myosin flow? If the anisotropic myosin flow seen in wild-type embryos requires tension to be maintained in directions parallel to the edge, cortical myosin loss in *dunk^1^* mutants may disrupt the interconnectedness of the actomyosin network and thereby release the tension that is necessary to constrain the direction of the flow. To test this paradigm, we generated a computer model to investigate the behavior of a two-dimensional, interconnected contractile network (Fig. 6A; supplementary Materials and Methods). Each edge of this virtual network contains multiple constricting parallel fibers connected by myosin nodes that resemble the puncta of cortical myosin as well as other cortical components that myosin associates with. The myosin nodes are initially spaced broadly to reflect the initial meshwork-like appearance of the actomyosin cytoskeleton (Fig. 1E). During simulation, the myosin nodes undergo dynamic turnover with a recruitment rate of *k_on_* and a dissociation rate of *k_off_* (see supplementary Materials and Methods for details). It is worth noting that *k_on_* and *k_off_* do not necessarily reflect the dynamics of cortical myosin turnover as measured in our FRAP experiments, because we do not distinguish whether changes in *k_on_* and *k_off_* are a result of alterations in the rate of the myosin recruitment and dissociation to and from its cortical binding sites, or a result of alterations in the availability of these sites. When *k_on_*≫*k_off_*, the number of myosin nodes is constant, and the network remains interconnected. As the model moves towards its minimum energy, the myosin nodes move anisotropically similar to the myosin flow observed in wild-type embryos. Within each edge, myosin nodes move perpendicular but not parallel to the edge, effectively reducing the width of edge without affecting the global architecture of the network (Fig. 6B,D; Movie 8; [*k_on_*=0.05, *k_off_*=0.001]).

As we reduced *k_on_*/*k_off_*, our model generated phenotypes that mimic the effect of Dunk loss. The final energy minimum configuration that best approximates the *dunk* phenotype is given by the parameters [*k_on_*=0.003, *k_off_*=0.001]. In this scenario, loss of myosin nodes generates local breaks within the network that cannot be reconnected promptly. As myosin continues to contract, the network ruptures, with most myosin nodes moving towards the neighboring vertices and becoming depleted from edges. As a result, the myosin nodes accumulate at discrete foci that are usually centered at vertices (Fig. 6C,E; Movie 8). Importantly, the biased vertex accumulation of myosin nodes results from unbiased distribution of myosin turnover dynamics within the contractile network, presumably as a result of the geometrical constraints of an interconnected network. Our simulation therefore demonstrates that the destabilized myosin flow and the resulting altered myosin distribution, in particular the accumulation of myosin at the vertices, can be a simple mechanical outcome of myosin loss that disrupts the tension balance within a contractile network.

### Dunk colocalizes with myosin at the invagination front during early and mid-cellularization

**Fig. 7.**
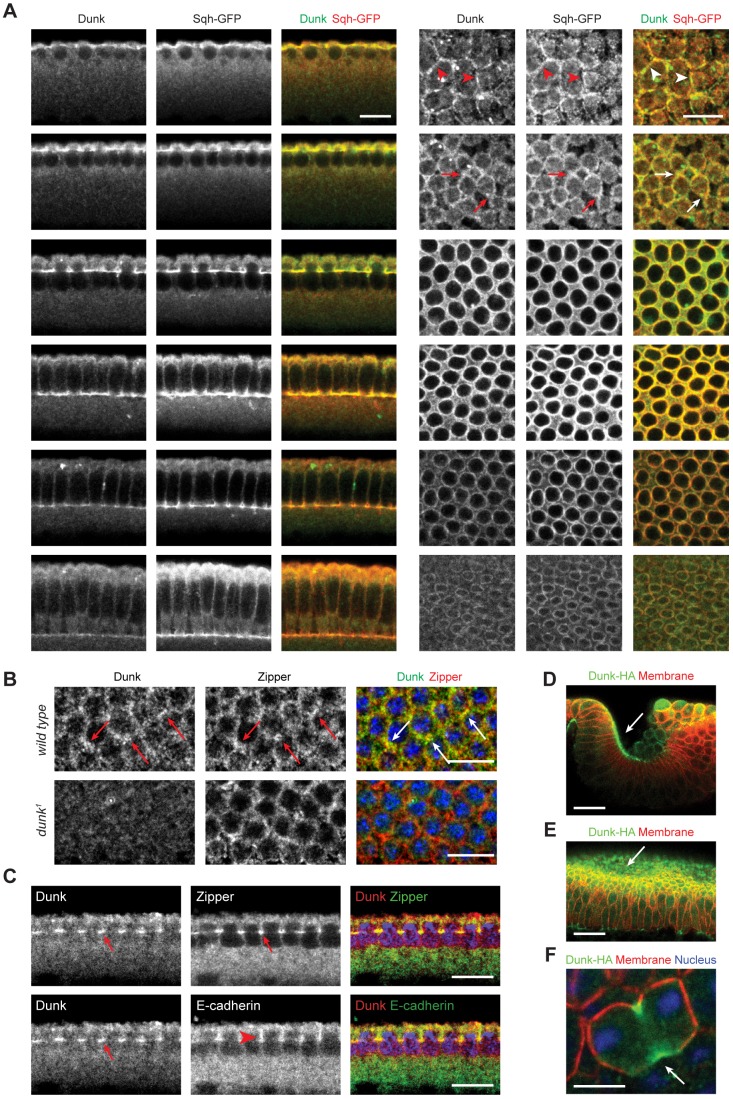
**Dunk colocalizes with myosin at the invagination front during early and mid-cellularization.** (A) Immunostaining of *sqh^AX3^; sqh-GFP* embryos with anti-Dunk and anti-GFP antibodies. Dunk co-localizes with Sqh-GFP at the invagination front during early and mid-cellularization but becomes undetectable at late cellularization. Left panels: mid-sagittal view; right panels: en face view. Arrowheads indicate the colocalization of Dunk and myosin at the old furrows surrounding the previous mitotic figure. Arrows indicate the enrichment of Dunk at the furrows between neighboring nuclei where myosin puncta are also enriched. Scale bars: 10 µm. (B) Immunostaining of wild-type (top) and *dunk^1^* mutant (bottom) embryos with anti-Dunk and anti-Zipper (MHC) antibodies. Arrows highlight the colocalization of Dunk and myosin as myosin becomes enriched at the furrows at the onset of cellularization. Scale bars: 10 µm. (C) Immunostaining of wild-type embryos with anti-Dunk, anti-Zipper and anti-DE-cadherin antibodies. Dunk and myosin colocalize at the invagination front (arrows); the basal adherens junctions localize immediately apical to Dunk and myosin (arrowhead). Scale bars: 10 µm. (D-F) Ectopically expressed Dunk-3HA localizes to contractile actomyosin structures (arrows) such as the apical actomyosin network in posterior midgut (D) or ventral furrow (E), and cytokinetic rings in dividing cells (F). Scale bars: 30 µm (D,E); 5 µm (F).

We generated polyclonal antibodies against full-length Dunk protein and examined its subcellular localization during cellularization. Dunk displays extensive colocalization with myosin at the invagination front throughout early to mid-cellularization (Fig. 7A). At the onset of cellularization, Dunk is first detected at the old furrows surrounding the previous mitotic figure (Fig. 7A, arrowheads), followed by an enrichment at the furrows between neighboring nuclei where myosin puncta are also enriched (Fig. 7A,B, arrows). Dunk localizes to the actomyosin rings during mid-cellularization but quickly becomes undetectable at late cellularization (Fig. 7A). Basal adherens junctions, as detected by staining of DE-cadherin (Shotgun), localize immediately apically to both myosin and Dunk (Fig. 7C, compare arrowhead and arrows). No specific protein signal was detected in *dunk^1^* mutant embryos when we stained them with the Dunk antibody, which confirmed that *dunk^1^ is* a protein null (Fig. 7B; Fig. S3).

We also made a rescue construct of *dunk*, in which the expression of full-length *dunk* coding sequence plus a C-terminal 3×HA tag is under the control of the *nullo* promoter (*P_nullo_*; Hunter et al., 2002). Dunk-3HA showed similar localization as endogenous Dunk and rescued the myosin phenotype in *dunk^1^* mutant embryos (Fig. S4).

### Ectopically expressed Dunk localizes to contractile actomyosin structures in post-blastoderm-stage embryos

*dunk* is not normally expressed in post-blastoderm-stage embryos. The rescue construct of *dunk* contains 14 *UAS* GAL4-binding sites upstream of *P_nullo_*, allowing us to express *dunk* ectopically at later stages using different GAL4 drivers. When expressed at post-blastoderm stages using maternally supplied GAL4 (67.15; Hunter and Wieschaus, 2000), Dunk-3HA invariably accumulates at locations where myosin is also enriched, such as the cytokinetic rings in the dividing cells and the apical cortex of apically constricting cells during the formation of ventral furrow and posterior midgut (Fig. 7D-F). These observations suggest that Dunk can be recruited to actomyosin contractile structures independently of other cellularization-specific gene products.

### Simultaneously eliminating *dunk* and *slam* disrupts both myosin flow and direct myosin recruitment

Despite the early myosin loss during the flow phase in *dunk^1^* mutant embryos, new myosin can still be recruited to the invagination front during the recruitment phase at a rate similar to that in wild-type embryos (Fig. 4E). This myosin recruitment replenishes myosin at the invagination front and allows the formation of the actomyosin network which eventually rearranges into individual rings (Fig. 4D; *t*=25 min). Although these rings are often less rounded and distorted in shape, they are capable of driving basal closure during late cellularization (data not shown). *dunk^1^* mutant embryos show no obvious defects in the rate of furrow ingression (Fig. S5), and subsequent development is normal.

**Fig. 8.**
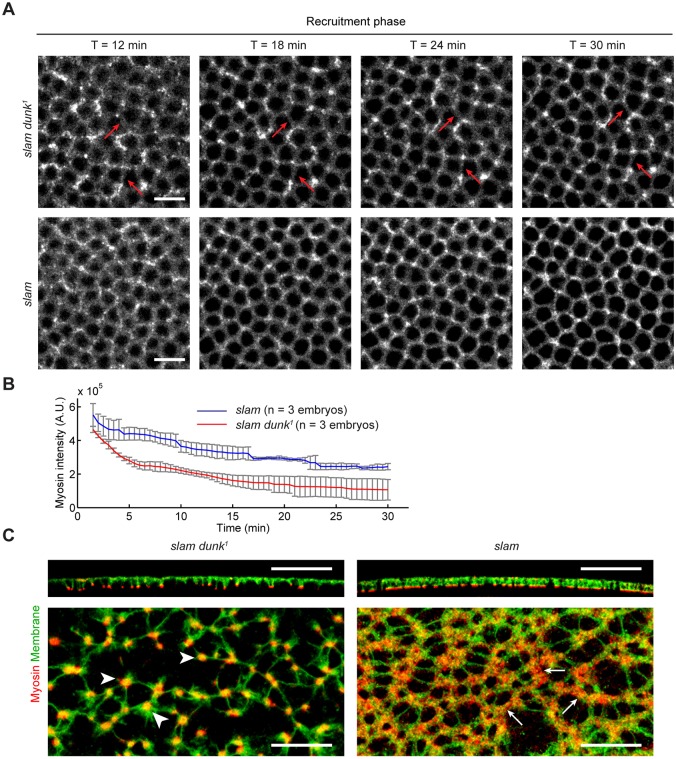
**Slam and Dunk function in different phases of myosin recruitment during cellularization.** (A) Projections of confocal sections showing Sqh-GFP at the invagination front (Sqh-GFP) in *slam* and *slam dunk^1^* mutant embryos during the recruitment phase. Arrows highlight edges that remain devoid of myosin throughout cellularization. Scale bars: 10 µm. (B) Quantification of total myosin intensity at the invagination front as percentage of intensity at *t*=2 min. Error bars indicate s.d. (C) Immunostaining showing localization of myosin (Zipper) and the plasma membrane marker Neurotactin in cross-sections (top) and projections of confocal sections at the invagination front (bottom) in *slam* and *slam dunk^1^* mutant embryos. Arrowheads highlight the discrete myosin foci in *slam dunk^1^* mutant embryos. Arrows highlight the partially connected actomyosin network in *slam* single mutant embryos. Scale bars: 50 µm (top); 25 µm (bottom).

What accounts for the Dunk-independent myosin recruitment during the recruitment phase? A possible candidate is Slam. Slam plays a crucial role in extension of the cleavage furrows during cellularization (Lecuit et al., 2002; Acharya et al., 2014). In *slam* mutants, the assembly of the basal-lateral surface at the invagination front is defective, and the rate of furrow ingression is greatly reduced. Interestingly, Slam has also been shown to promote the accumulation of myosin at the invagination front (Lecuit et al., 2002; Acharya et al., 2014). Slam directly binds to RhoGEF2 and is required for recruitment of RhoGEF2 to the invagination front (Wenzl et al., 2010). RhoGEF2 is a guanine nucleotide exchange factor for Rho1 and has been shown to promote actin assembly and myosin activation at the invagination front through Rho1 and its downstream effectors (Crawford et al., 1998; Padash Barmchi et al., 2005; Grosshans et al., 2005). In order to test whether Slam is required for new myosin recruitment during the recruitment phase, we examined *slam dunk* double mutant embryos and compared them with individual single mutants. In the double mutant, the increase in myosin intensity during the recruitment phase seen in both wild-type and *dunk* mutant embryos is completely abolished (Fig. 8A,B). The edges where myosin is lost during the flow phase remain devoid of myosin throughout cellularization (Fig. 8A, arrows). As a result, myosin never forms an interconnected network and the subsequent reorganization into individual contractile rings completely fails (Fig. 8A; Movie 9). In *slam* single mutants, myosin intensity barely increases during the recruitment phase (Fig. 8A,B), yet the actomyosin network remains partially connected (Fig. 8C, arrows). This is in contrast to the *slam dunk* double mutant in which the actomyosin network completely breaks down into discrete foci (Fig. 8C, arrowheads). Therefore, the Dunk-dependent stabilization of myosin during the flow phase and the Slam-dependent new myosin recruitment synergistically contribute to actomyosin organization at the invagination front.

Overall, our results suggest that cortical myosin flow and direct myosin recruitment are separately regulated by Dunk and Slam during cellularization. Whereas the flow phase requires Dunk, the recruitment phase is Dunk independent but requires Slam. It is worth noting that *slam* mutant embryos also show an overall reduction in cortical myosin intensity shortly after the onset of cellularization (Fig. 8B). It is conceivable that this early myosin loss is an indirect effect of the defects in the assembly of the basal-lateral surface previously seen in *slam* mutant embryos (Lecuit et al., 2002; Acharya et al., 2014). However, we could not formally exclude the possibility that Slam functions in both phases of myosin recruitment.

## DISCUSSION

Mechanisms regulating the recruitment and maintenance of myosin at the cell cortex play a crucial role in actomyosin-mediated force generation, which drives many morphogenetic processes. During *Drosophila* cellularization, 5000 contractile actomyosin rings are formed simultaneously with great temporal and spatial precision to drive the cleavage of the syncytial blastoderm. In this study, we found that myosin is recruited to the cellularization front by two distinct mechanisms that act at consecutive phases. In the flow phase (*t*=0-12 min), myosin recruited to the apical cortex rapidly flows towards the base of the newly formed furrows while the intensity of cortical myosin remains constant. In the recruitment phase (*t*=12-30 min), more myosin is directly recruited to the leading edge without undergoing cortical flow (Fig. 1I,J). Both cortical flow and direct recruitment have been implicated in the recruitment of myosin to the equatorial cortex during animal cytokinesis, but the timing and extent of their involvement remain unclear. Our results for the first time show that the two mechanisms can be used sequentially in a cytokinetic process.

In addition, we show that these distinct phases are under separate developmental regulation by transcription of different zygotic genes. *Drosophila* cellularization occurs coincident with a key developmental transition called mid-blastula transition (MBT), which is characterized by activation of zygotic gene expression (Edgar and Schubiger, 1986). We identified *dunk*, which we show to be transcriptionally activated immediately before cellularization (Fig. 3) and to be required specifically during the flow phase to maintain myosin at the cortex. In *dunk* mutant embryos, myosin rapidly dissociates from the cortex after the onset of the flow phase (Fig. 4D-F). Residual myosin preferentially flows towards the vertices and causes nearly complete depletion of myosin from edges (Fig. 5A,B). In the recruitment phase, however, myosin replenishes at the invagination front through a Slam-dependent but Dunk-independent, direct myosin recruitment pathway (Fig. 4D-F; Fig. 8). *Drosophila* cellularization therefore provides an example of a process in which the independent mechanisms of cortical myosin flow and direct myosin recruitment are used in separate phases with distinct genetic regulation.

The identification of two phases of myosin recruitment also enables us to define the Dunk-dependent cellular mechanism that regulates myosin flow. The flow of myosin immediately after the onset of cellularization is similar to the tension-driven myosin flow observed in *Dictyostelium*, *Drosophila* and mammalian cells during cytokinesis (Yumura et al., 2008; Uehara et al., 2010; DeBiasio et al., 1996). Using laser ablation, we demonstrate that the invagination front is under tension (Fig. 2). The build-up of tension is coincident with myosin recruitment to the cortex and probably results from the intrinsic contractility of the actomyosin structures induced by spatially restricted Rho1 activation near the equatorial cortex (Green et al., 2012). Geometric constraints at the invagination front further define the tension balance across the actomyosin network and impose an anisotropy on the myosin constrictions such that myosin flow mainly occurs perpendicular to the edges. We propose that Dunk-dependent stabilization of myosin recruitment sites enables the establishment of an interconnected actomyosin network that maintains tension in directions parallel to the edges, thereby providing a mechanical mechanism to guide the anisotropic myosin flow. In the absence of Dunk, the loss of cortical myosin disrupts the interconnectedness of the actomyosin network and causes myosin to flow parallel to the edge. In support of this paradigm, our computer simulation demonstrates that the observed effect of Dunk loss on myosin flow, in particular the biased flow towards vertices, could be a simple mechanical consequence of reducing myosin from the cortex (Fig. 6). Our results suggest that maintaining myosin levels at the cortex is a general requirement for reinforcing the mechanical stability and directionality of the actomyosin flow, although in different systems the specific molecular players might be different. It will be of interest to compare the mechanism used in *Drosophila* cellularization with those employed in other processes, such as the cortical actomyosin flow in *Caenorhabditis elegans* embryos during polarity establishment (Munro et al., 2004) and yolk actomyosin flow during zebrafish epiboly (Behrndt et al., 2012).

Previous experiments reducing actomyosin contractility argue against a major role of basal contractility in the rate of furrow ingression during cellularization (Royou et al., 2004; Thomas and Wieschaus, 2004). Our analysis of the *dunk* mutants, however, reveals an important role of the actomyosin network in regulating basal morphology. When tension balance is lost, myosin flow is redirected to the axis parallel to the edge. As myosin flows away from the edge, the edge elongates. Conversely, as myosin becomes concentrated at the edge, the edge shortens (Fig. 5A-G). In extreme cases, the shortening of the edges causes the neighboring vertices to merge, resulting in abnormal basal morphology with an increase of quadrilaterals at the cost of hexagons and pentagons (Fig. 5H). As a consequence, the actomyosin rings later formed at the invagination front also acquire irregular shapes (Fig. 4A). Our findings suggest that actomyosin contractility prior to the formation of discrete cytokinetic rings may serve the following functions: (1) enrichment of myosin at the invagination front through cortical flow, (2) alignment of an initially less organized meshwork of actomyosin filaments into arrays parallel to the edge, and (3) maintenance of tension balance at the invagination front as a mechanism for regulating the hexagonal packing of the ingressing furrows.

Actomyosin-based contractility has been widely implicated in a variety of morphogenetic processes (Munjal and Lecuit, 2014; Martin and Goldstein, 2014). It is crucial to understand how myosin is recruited to the right place where forces are generated and how developmental control of this recruitment regulates force generation and the resulting tissue mechanics. Because cellularization in *Drosophila* allows us to distinguish temporally the cortical flow and direct recruitment mechanisms thought to play roles in most cytokinetic processes, it offers an advantageous system in which to investigate these mechanisms separately. Our identification of zygotic genes that independently regulate each phase also provides a unique opportunity to study the interplay between cell signaling, actomyosin organization and tissue mechanics during a morphogenetic process.

## MATERIALS AND METHODS

### Fly stocks and genetics

*OreR* embryos were used as a control for *in situ* hybridization and immunostaining experiments unless stated otherwise. The *dunk^1^* P-element insertion mutant line, *P{SUPor-P}CG42748^KG09309^* (Bellen et al., 2004), was obtained from Bloomington *Drosophila* Stock Center. The *slam* mutant embryos were generated using a *Df(2L)dpp[s7-dp35] 21F1–3;22F1–2 (halo) Df(2L)Exel6016(slam)/Cyo sqh-GFP* line. The *slam dunk* double mutant embryos were generated using a *Df(2L)dpp[s7-dp35] 21F1–3;22F1–2 (halo) Df(2L)Exel6016(slam) P{SUPor-P}CG42748^KG09309^(dunk^1^)/Cyo sqh-GFP* line as previously described (He et al., 2014).

For ectopic expression of Dunk in post-blastoderm-stage embryos, *UAS-dunk-3×HA (II); UAS-dunk-3×HA (III)* females were crossed to males from the Maternal-Tubulin-Gal4 line 67.15 (Hunter and Wieschaus, 2000) to generate *UAS-dunk-3×HA (II)*/*Maternal-Tubulin-Gal4 (II)*; *UAS-dunk-3×HA (III)*/*Maternal-Tubulin-Gal4 (III)* flies. Embryos derived from these flies were used to examine the localization of the ectopically expressed Dunk-3×HA.

The dynamics of myosin in wild-type and *dunk^1^* mutant embryos was monitored in embryos from the *yw sqh^AX3^; sqhGFP* stock (Royou et al., 2002) and the *dunk^1^; sqhGFP* stock (this study), respectively. *sqh* encodes *Drosophila* regulatory light chain of non-muscle Myosin II (Karess et al., 1991).

### Generation of *dunk*-rescuing construct

For phenotypic rescue experiments, a fusion DNA containing (from 5′ to 3′) the *nullo* promoter (*P_nullo_*=498 bp sequence upstream of the *nullo* CDS), *dunk* (*CG34137*) CDS and a sequence encoding a 3×HA tag was synthesized by GenScript USA and was subsequently inserted into a transformation vector containing the attB site (pTiger, courtesy of S. Ferguson, State University of New York at Fredonia, Fredonia, NY, USA). The vector also contains 14 *UAS* GAL4-binding sites upstream of *P_nullo_*, allowing us to express *dunk* ectopically at later stages using different GAL4 drivers. The resulting construct was sent to Genetic Services for integration into the attP40 and attP2 site using the phiC31 integrase system (Groth et al., 2004). Dunk-3×HA expressed under the control of *P_nullo_* shows similar spatial distribution and temporal pattern as the endogenous Dunk.

### Generation of Dunk antibody

An N-terminal His-tagged Dunk full-length fusion protein was expressed in *Escherichia coli* and purified by GenScript USA. Purified antigen was injected into rats and guinea pigs by a commercial supplier (Panigen). Raw serum was used for immunostaining of fixed embryos. No specific protein signal was detected at the invagination front in dunk mutant embryos when stained with the Dunk antibody.

### Embryo fixation, antibody staining and *in situ* hybridization

Antibody staining against myosin (Zipper), Armadillo and Neurotactin was performed on heat-fixed embryos. Antibody staining against Dunk, Rho1 and Dunk-3×HA was performed on formaldehyde-fixed embryos. The vitelline membrane was removed by shaking in heptane and methanol after fixation. For phalloidin staining, embryos were formaldehyde fixed, and the vitelline membrane was removed by shaking in heptane and ethanol after fixation. Embryos were blocked with 10% bovine serum albumin (BSA) in PBS and 0.1% Tween 20, and incubated with primary antibodies in PBT (PBS/0.1% BSA/0.1% Tween 20) overnight at 4°C at the following dilutions: rabbit anti-Zipper 1:100 (Sokac and Wieschaus, 2008b); monoclonal mouse anti-Armadillo 1:50 (N2 7A1, Developmental Studies Hybridoma Bank); monoclonal rat anti-DE-cadherin 1:25 (DCAD2, Developmental Studies Hybridoma Bank); monoclonal mouse anti-Neurotactin 1:10 (BP 106, Developmental Studies Hybridoma Bank); rabbit anti-GFP 1:500 (EMD Millipore); rat or guinea pig anti-Dunk 1:500; and rat anti-HA 1:100 (Roche Life Science). Secondary antibodies coupled to Alexa Fluor 488, Alexa Fluor 561 and Alexa Fluor 647 were used at 1:500 (Invitrogen). Embryos were mounted in Aqua Poly Mount (Polysciences) for confocal imaging. Confocal images were collected on a Leica SP5 confocal microscope with a 63×/1.3 NA glycerine-immersion objective lens and a pinhole setting of 1 airy unit.

*In situ* hybridization was performed following standard procedures (Tautz and Pfeifle, 1989) using a 0.7 kb antisense RNA probe to *dunk*. The RNA probe was generated by in vitro transcription using the following primers to produce the DNA template: gcggatccatgtcagcattcacctgcacacag and taatacgactcactatagggtatgctcagccgaccttttt.

### Live imaging

To prepare embryos for live imaging, manually staged embryos expressing Sqh-GFP were collected at room temperature (22-25°C) on agar plates, dechorionated in 50% bleach for 2-4 min, rinsed thoroughly with water, and transferred on a 35 mm MatTek glass-bottom dish (MatTek Corporation). Distilled water was then added to the dish well to completely cover the embryos. All imaging was performed in water at room temperature.

For quantification of Sqh-GFP intensities, Sqh-GFP videos were obtained on a Leica SP5 confocal microscope with a 63×/1.3 NA glycerin-immersion objective lens. A 5× zoom was used. Fifteen confocal *z*-sections with a step size of 1 µm were acquired every 12 s. The image size was 512×512 pixels, which corresponds to a lateral pixel size of 96 nm. The total imaged volume is approximately 49×49×14 µm.

For 3D reconstruction of myosin structures, Sqh-GFP videos were obtained on a Leica SP5 confocal microscope with a 100×/1.4 NA oil-immersion objective lens. A 5× zoom was used. Twelve confocal *z*-sections with a step size of 0.5 µm were acquired every 4.2 s. The image size was 512×256 pixels, which corresponds to a lateral pixel size of 60.5 nm. The total imaged volume is approximately 31×15×5.5 µm. It is worth noting that this imaging approach (high magnification and fast frame rate, which is necessary to reveal the morphological details in 3D reconstruction and the fast dynamics of the myosin flow) causes photobleaching over time. For this reason, for experiments in which we quantify the cortical myosin intensity, we imaged the sample with lower magnification and slower frame rate to minimize photobleaching. The latter approach, however, does not provide sufficient resolution (both temporal and spatial) to visualize details of the myosin flow.

### Image analysis, quantification and statistics

For quantification of myosin fluorescence intensity at the invagination front, the Sqh-GFP movies were analyzed using MATLAB (Image Processing Toolbox, The MathWorks, Natick, MA, USA) as follows. First, images were subject to background subtraction. The background is defined as the cytoplasm level of Sqh-GFP at regions right below the nuclei at the beginning of cellularization. Second, background-corrected images from five adjacent confocal cross-sections (∼4 µm thick) covering the entire invagination front were summed. Third, total intensity was calculated from the summed image at each time point. Finally, the signal was normalized between embryos according to the intensity of cytoplasmic Sqh-GFP.

To compare myosin fluorescence intensity at the edges and vertices, the Sqh-GFP movies were analyzed as follows. Images were subject to background subtraction and summed for five slices, which covers the entire invagination front as mentioned above. To define signals that belong to edges versus vertices, the basal outline of the cells (as marked by Sqh-GFP) were segmented using the MATLAB-based software package Embryo Development Geometry Explorer (EDGE; Gelbart et al., 2012). In EDGE, the outlines of individual cells are represented by polygons and tracked over time. Along each polygon, we define points less than 1.2 µm away from the nearest vertex as ‘vertex’, and points more than 1.2 µm away from the nearest vertex as ‘edge’. Mean intensity was integrated at vertices and edges along the corresponding line segments with a width of 0.6 µm. The intensity was then normalized between embryos according to the intensity of cytoplasmic Sqh-GFP. To measure the correlation between edge length and myosin intensity, a correlation coefficient was calculated between the edge length and the mean Sqh-GFP intensity along the edge per time point per embryo.

For 3D reconstruction of myosin structures, image stacks of Sqh-GFP were deconvoluted using the ImageJ (NIH) plugin ‘Iterative Deconvolve 3D’ (http://www.optinav.com/Iterative-Deconvolve-3D.htm). 3D point spread function (PSF) generated by the ImageJ plugin ‘Diffraction PSF 3D’ (http://www.optinav.com/Diffraction-PSF-3D.htm) based on diffraction theory was used in deconvolution. The deconvolved image stacks were then subject to 3D rendering using NIS-Elements (Nikon Instruments).

To measure the rate of furrow ingression, kymographs were generated from Sqh-GFP movies using MATLAB, and the depth of the invagination front from the surface of the embryos over time was manually measured using ImageJ.

### Laser ablation

Sqh-GFP embryos were prepared for live imaging and were imaged using a spinning disk confocal microscope (Ultraview; PerkinElmer) with a 60×/1.4 NA oil-immersion objective (Nikon), a 488-nm laser, and an electron-multiplying charge-coupled device camera (C9100-13; Hamamatsu). The microscope was controlled with Volocity acquisition software (Improvision). Ablation was performed using a Micropoint laser (Andor Technology) tuned to 365 nm. For each ablation, a focused laser beam was targeted to the middle of an edge marked by Sqh-GFP to generate a point incision. Time-lapse movies of a single *z*-slice focused at the level of the invagination front were acquired immediately before and after ablation to measure the movement of surrounding tissues upon release of tension. As a control, ablation was performed at the apical cortex in embryos at cycle 13 anaphase.

Velocity maps of myosin flow during the flow phase and tissue movement immediately after laser ablations were generated using the MATLAB-based software OpenPIV (Taylor et al., 2010) with a spacing/overlap of 8×8 pixels and an interrogation window size of 32×32 pixels. For the laser-ablation experiments, average velocity map was generated from 24 ablations in eight embryos at approximately 5 min after the onset of cellularization.

### FRAP analysis of cortical myosin turnover

Sqh-GFP embryos at early cellularization were prepared for live imaging and were imaged on a Leica SP5 confocal microscope with a 63×/1.3 NA glycerin-immersion objective lens. A 5× zoom was used. Photo-bleaching was performed on a single *z*-slice focused at the level of the invagination front. A rectangular region (∼35 µm×10 µm) was bleached using the 458, 476, 488 and 496 lines from the argon laser operating at 75% laser power. Fifteen iterations were used for bleaching, which lasted approximately 5.5 s. Six confocal *z*-sections with a step size of 1 µm, which spans the invagination front, were acquired every 2.35 s before and after photobleaching. The image size was 512×512 pixels, which corresponds to a lateral pixel size of 96 nm. The total imaged volume was approximately 49×49×5 µm.

To acquire the rate of fluorescence recovery, we analyzed the FRAP movies using MATLAB as follows. For each time point, we generated weighted sum from all six *z*-slices after background subtraction. Background was defined as 50% of the cytoplasmic level of Sqh-GFP. We used 50% rather than 100% of the cytoplasmic intensity as background in order to capture the initial recovery of the cortical signals. The weight for each slice was proportional to the Sqh-GFP signal from the unbleached control region after subtracting the cytoplasmic signal. We then measured the fluorescence intensities from the summed images for both the bleached region and the control unbleached region. The fluorescence intensities were normalized to between 0 and 1 for each region. To acquire the half recovery time *t_1/2_*, we took the ratio between the intensities measured within the bleached region and the control region from the same embryo. In both wild-type and *dunk* mutant embryos, the bleached region becomes nearly fully recovered as far as the myosin loss is compensated for. We therefore defined the time when the ratio reaches 0.5 as the half recovery time. Measurements from seven wild-type and 16 *dunk^1^* mutant embryos were analyzed, and the average *t_1/2_* was reported.

### Computer simulation of a contractile network

In order to demonstrate how tension drives anisotropic myosin flow and how myosin turnover at the cortex affect the mechanics of the network, we generated a computer model to simulate the behavior of an interconnected contractile network. See supplementary Materials and Methods for full details of the model and source code for image analysis and computational modeling.
